# *ccdC* Regulates Biofilm Dispersal in *Bacillus velezensis* FZB42

**DOI:** 10.3390/ijms25105201

**Published:** 2024-05-10

**Authors:** Lin Shao, Zizhu Shen, Meiju Li, Chenyun Guan, Ben Fan, Yunrong Chai, Yinjuan Zhao

**Affiliations:** 1 Co-Innovation Center for Sustainable Forestry in Southern China, College of Forestry and Grass, Nanjing Forestry University, Nanjing 210037, China; 2 College of Life Science, Nanjing Forestry University, Nanjing 210037, China; 3 Department of Biology, Northeastern University, Boston, MA 02115, USA

**Keywords:** *Bacillus velezensis* FZB42, biofilm dispersal, *ccdC*, motility, c-di-GMP

## Abstract

*Bacillus velezensis* FZB42 is a plant growth-promoting rhizobacterium (PGPR) and a model microorganism for biofilm studies. Biofilms are required for the colonization and promotion of plant growth in the rhizosphere. However, little is known about how the final stage of the biofilm life cycle is regulated, when cells regain their motility and escape the mature biofilm to spread and colonize new niches. In this study, the non-annotated gene *ccdC* was found to be involved in the process of biofilm dispersion. We found that the *ccdC*-deficient strain maintained a wrinkled state at the late stage of biofilm formation in the liquid—gas interface culture, and the bottom solution showed a clear state, indicating that no bacterial cells actively escaped, which was further evidenced by the formation of a cellular ring (biofilm pellicle) located on top of the preformed biofilm. It can be concluded that dispersal, a biofilm property that relies on motility proficiency, is also positively affected by the unannotated gene *ccdC.* Furthermore, we found that the level of cyclic diguanylate (c-di-GMP) in the *ccdC*-deficient strain was significantly greater than that in the wild-type strain, suggesting that *B. velezensis* exhibits a similar mechanism by regulating the level of c-di-GMP, the master regulator of biofilm formation, dispersal, and cell motility, which controls the fitness of biofilms in *Pseudomonas aeruginosain.* In this study, we investigated the mechanism regulating biofilm dispersion in PGPR.

## 1. Introduction

It is now believed that microorganisms often grow at interfaces to form polymicrobial aggregates such as films, mats, flocs, sludge, or ‘biofilms’ [1]. These microorganisms are critical for bacterial survival, adaptation, and dissemination in natural, industrial, and medical systems [2,3]. Biofilms represent a protected life form that enables bacteria to survive in hostile environments [1]. Sessile cells are embedded in self-produced complex polysaccharides, proteins, lipids, and extracellular DNA, collectively referred to as extracellular polymeric substances (EPSs) [4,5]. This is a developmental process that is initiated by surface attachment by planktonic cells. As the biofilm ages, the population of cells becomes stressed due to overcrowding, nutrient restriction, and waste product accumulation. Dispersion is the terminal stage of biofilm development, during which bacteria evacuate a mature biofilm and transition to a planktonic state.

During active dispersion, bacteria actively initiate mechanisms in response to a(n) (external) trigger, usually an environmental change, which results in the release of cells into the environment [6]. One of the underlying mechanisms is the active degradation of the secondary messenger cyclic diguanosine monophosphate (c-di-GMP), also called cyclic diguanylate, which is a major regulatory component in both biofilm development and dispersion [7]. Low intracellular c-di-GMP concentrations promote a planktonic lifestyle, while high concentrations stimulate biofilms.

A decrease in the c-di-GMP concentration activates the expression of genes involved in motility and genes involved in matrix degradation [8]. Several pathogenic bacteria, including *Pseudomonas aeruginosa*, have been investigated for their ability to disperse biofilms, as the induction of biofilm dispersion could be a potential strategy to help combat biofilm-related infections. Plant growth-promoting bacteria, such as *Bacillus* species, have been investigated in terms of their ability to form biofilms and colonize roots, but their role in the process of biofilm dispersion is not well understood. However, surprisingly, it was shown that dispersed *P. aeruginosa* cells are distinct from biofilm cells and planktonic cells. In addition, dispersed cells have lower c-di-GMP concentrations than planktonic cells. Lower c-di-GMP concentrations have been linked to increased virulence, suggesting that dispersed cells are more virulent than planktonic cells [9]. Indeed, virulence assays confirmed that dispersed cells were more effective at penetrating and killing macrophages than their planktonic counterparts [10]. Moreover, dispersed cells appeared to be more effective at killing *Caenorhabditis elegans* than planktonic cells [10]. These results indicate that dispersed cells, which have the potential to disseminate through the body, can worsen clinical outcomes. Indeed, in vivo studies have described the spread of infection following dissemination events [11,12]. The results clearly indicated that dispersion is complex, that caution should be exercised when using the biofilm dispersal strategy, and that further data on the susceptibility of the dispersed cells are required before this approach is introduced into clinical practice. Therefore, it is not yet clear whether biofilm dispersion in plant growth-promoting bacteria affects biofilm application in the rhizosphere. What impact does this stage have on the growth-promoting and stress-resistant effects of the rhizosphere?

*Bacillus velezensis* FZB42 is a Gram-positive, endospore-forming bacterium that can promote plant growth with potent biocontrol activity [13,14]. It was isolated from the inter-root of German sugar beet in 1998 and was the first Gram-positive biocontrol bacterium whose entire genome was sequenced [14,15]. The genome analysis of *B. velezensis* FZB42 revealed that nearly 10% of the FZB42 genome is devoted to synthesizing antimicrobial metabolites and their corresponding immunity genes [13]. As a result, researchers have focused more on antibiotics than biofilms. Another important PGPR is the Gram-positive soil bacterium *Bacillus subtilis*, which is a well-studied species with robust biofilm-forming capabilities, which has made it an ideal model for biofilm research. *B. velezensis* FZB42 is highly homologous to *B. subtilis* 168 [14]. *B. subtilis* compromises a complex system to regulate biofilm formation against extended harsh conditions in the natural environment [16,17]. The *tapA-sipW-tasA* operon and the *epsA-O* operon, which encode the biofilm matrix, are regulated indirectly by the response regulator protein Spo0A [18]. The activity of Spo0A is controlled by at least four sensor histidine kinases: KinA, KinB, KinC, and KinD [19]. Although the regulatory pathway for biofilm formation has been reported in *B. subtilis*, it has hardly been studied in other *Bacillus* species [20].

The molecular mechanisms that regulate biofilm dispersal in Gram-positive bacteria are poorly understood. It has been established that the transcription factors SigB and SinR control whether cells remain in or leave a biofilm when metabolic conditions become unfavorable in *B. subtilis* [21]. However, when it comes to *B. velezensis*, there have been no investigations on biofilm dispersion. Here, we identified a novel gene, *ccdC*, that controls biofilm dispersal in *B. velezensis* FZB42. We showed that *ccdC* regulates motility, sporulation, matrix degradation, and other behaviors during dispersion. We also hypothesize that the level of c-di-GMP influences dispersal in *B. velezensis*.

## 2. Results

### 2.1. The ccdC Gene Regulates Biofilm Development in B. velezensis FZB42

**Figure 1:**
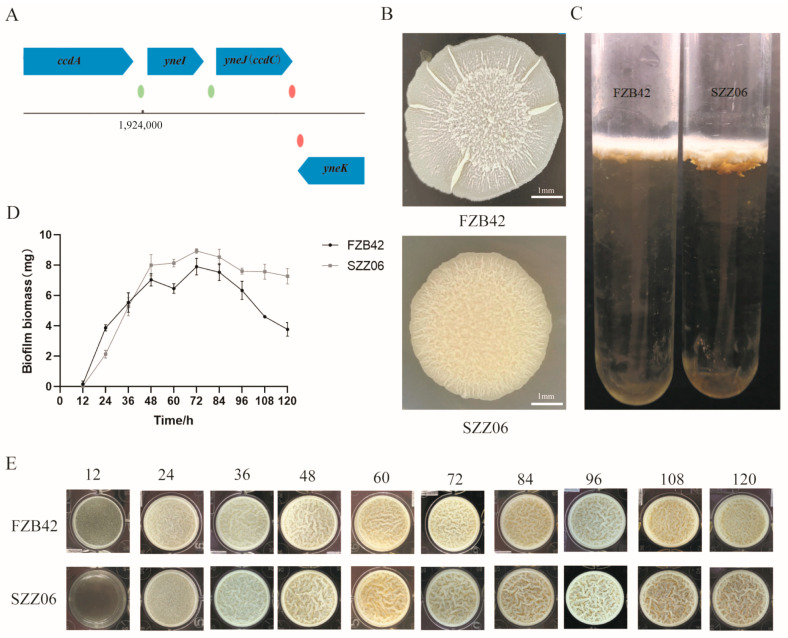
*ccdC* regulates biofilm development. (**A**) *ccdC* (*yneJ*) genomic context in *B. velezensis*. (**B**,**C**) Biofilm formation of FZB42 and SZZ06 on solid surfaces (**B**) and at air–liquid interfaces (**C**) at 48 h. (**D**) Dry weight of pellicle biofilms of FZB42 and SZZ06 collected from (**E**). (**E**) Pellicle biofilm development of FZB42 and SZZ06 over a period of 120 h.

We screened a transposon insertion mutant library in *B. velezensis* FZB42 for altered biofilm phenotypes. For one such mutant (named SZZ06), we mapped the transposon insertion site in the *ccdC* (formerly known as *yneJ*) gene (FZB42 *ccdC*::*TnYLB-1*). *ccdC* is located downstream of *ccdA* and immediately downstream of *ccdB* in the genome of *B. velezensis* (Figure 1A). There are no functional annotations or reports of the *ccdC* gene in subtiwiki’s study [22]. ORF160 (also known as the *yneJ* open reading frame) is homologous to both open reading frames ORF120 and ORF160, which were found to be cotranscribed with *ccdA*. Biofilms of wild-type FZB42 and mutant SZZ06 were compared (Figure 1). Compared with wild-type FZB42, the colony biofilm of SZZ06 had a completely different appearance on the solid surface (Figure 1B). FZB42 had a circular area in the center of the biofilm, which was ring-shaped and raised, with distinct irregular dot-like protrusions within the circular area. However, the biofilm of SZZ06 had no clear area delineation and appeared wrinkled as a whole. When pellicle biofilms formed at the air–liquid interface, SZZ06 formed thicker and more wrinkled biofilms than FZB42 (Figure 1C). We measured pellicle biofilm development after treatment with FZB42 and SZZ06 for a total of 120 h. We found that in the early stage of biofilm assembly, SZZ06 had a delayed pellicle biofilm appearance compared with FZB42 (Figure 1E). After continued incubation, the pellicle biofilms of SZZ06 became thicker, and the dry weight of the biofilms became greater (Figure 1D,E). After 72 h, although FZB42 and SZZ06 still maintained a mature biofilm morphology, the dry weight of both strains started to decrease (Figure 1D,E). When the incubation time further increased to 108 h, the FZB42 biofilms started to collapse gradually, while the SZZ06 biofilms still maintained relatively intact morphology (Figure 1D,E). Overall, our results suggest that the disruption of the *ccdC* gene caused a delay in the aging and dispersal of biofilms in *B. velezensis* FZB42.

### 2.2. ccdC Is More Highly Expressed in Mature Biofilms Than in Early Biofilms

**Figure 2:**
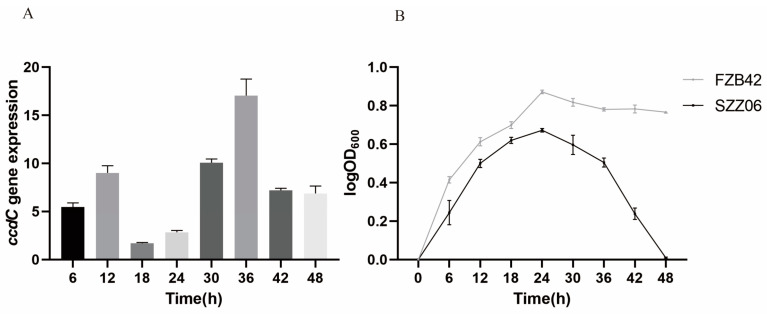
*ccdC* and its role in biofilm life cycles. (**A**) *ccdC* gene expression level in FZB42 during incubation every 6 h. (**B**) Growth curves of FZB42 and SZZ06 during 0–48 h.

We hypothesized that *ccdC* is involved in the regulation of the biofilm development cycle in *B. velezensis* FZB42. We sought to first determine whether and when *ccdC* is induced during the biofilm development cycle. To test this hypothesis, we quantified the relative expression level of *ccdC* by real-time PCR and analyzed *ccdC* expression in cells collected at different time points during biofilm development (logarithmic growth, biofilm assembly, biofilm aging, and biofilm dispersal) (Figure 2A). *ccdC* gene expression showed a “wave-like” pattern during the 48 h biofilm development cycle; its expression was lowest at 18 h (the biofilm assembly phase) and highest at 36 h (the biofilm dispersal phase). The results suggest that there are more *ccdC*^OFF^ cells in the mature biofilm and more *ccdC*^ON^ cells when the biofilm is dispersed. The *ccdC* gene appears to be strongly induced during biofilm dispersal.

We also wondered whether the disruption of *ccdC* impairs *B. velezensis* growth. Thus, the growth curves of FZB42 and SZZ06 were compared at 37 °C and 200 rpm (Figure 2B). Both FZB42 and SZZ06 entered the stationary phase at 24 h. However, before entering the stationary period, SZZ06 grew more slowly than wild-type FZB42 during the same period, and after entering the stationary stage, SZZ06 declined at a faster rate. At 48 h, the OD value of SZZ06 was close to 0. These results indicate that *ccdC* plays an important role in both the biofilm cycle and growth phase.

### 2.3. ccdC Is Essential for Biofilm Dispersal

**Figure 3:**
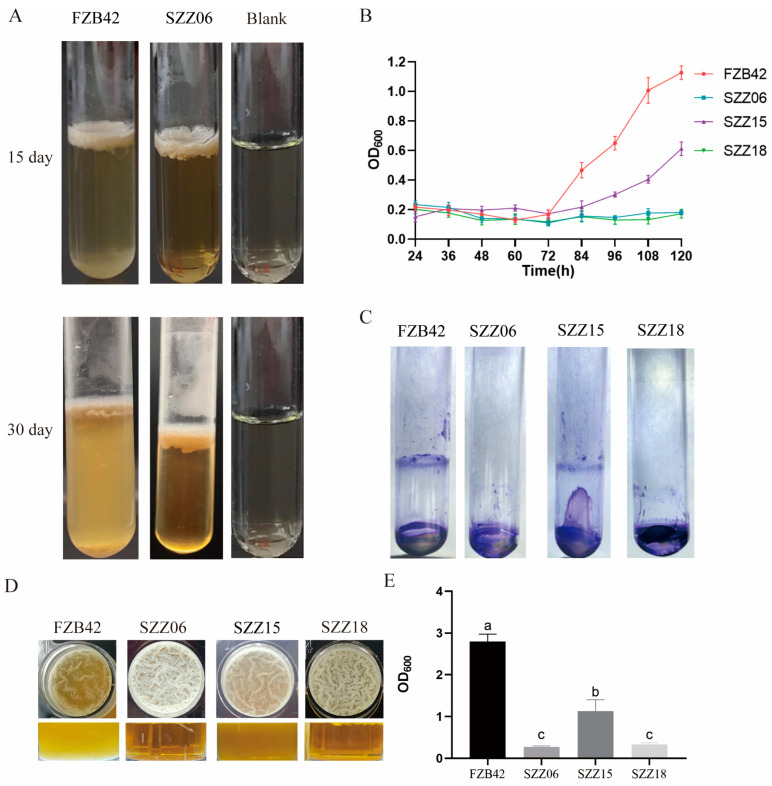
*ccdC* is essential for biofilm dispersion. (**A**) Biofilm downward dispersion of FZB42 and SZZ06 in LBGM. (**B**) Changes in the OD_600_ of the bottom solution of the FZB42, SZZ06, SZZ15, and SZZ 18 biofilms during 0–120 h. (**C**) Biofilm upward dispersion of FZB42, SZZ06, SZZ15, and SZZ18 (crystal violet staining). (**D**) FZB42, SZZ06, SZZ15, and SZZ18 in the final stage of biofilm dispersion. (**E**) Changes in the OD_600_ of the bottom solution of the FZB42, SZZ06, SZZ15, and SZZ18 biofilms on the 10th day. *t*-test was performed, where Different letters indicate the significant difference at *p* = 0.05 level.

Disruption of the *ccdC* gene leads to severe cell death in the late stages of growth, yet it increases biofilm biomass during biofilm development (Figure 1D and Figure 2B). These two results seem to contradict each other. We hypothesized that *ccdC* is involved in the regulation of biofilm dispersal. In other words, the absence of *ccdC* in SZZ06 prevented the biofilm from dispersing properly, thus maintaining the biofilm biomass. At the end of biofilm growth, when the individual bacterial cells involved in dispersal reach a certain number, they are released from the matrix-encased biofilms into the external environment; therefore, the turbidity of the solution at the bottom of the biofilm can be observed and used to reflect the dispersal ability of the bacteria to a certain extent. We first observed the biofilm dispersal of FZB42 and SZZ06 in LBGM broth (Figure 3A). Under 25 °C static incubation conditions, the solution at the bottom of the FZB42 biofilm appeared slightly cloudy, while the solution at the bottom of the SZZ06 biofilm appeared clear. As the incubation time increased to 30 days, the turbidity of the solution at the bottom of the FZB42 biofilm gradually increased, while that of the solution at the bottom of the SZZ06 biofilm remained clear, and this phenomenon remained unchanged until the SZZ06 biofilm disintegrated (Figure 3A).

To evaluate the effects of the loss in *ccdC* on biofilm development and exclude possible point mutations during transposition, we reconstructed knockout and complementation strains of the *ccdC* gene and then compared the biofilm dispersal of FZB42 with that of SZZ06, SZZ15 (SZZ06 *amyE*::*ccdC*, *emR*), and SZZ18 (FZB42Δ*ccdC*::*speR*) by measuring the OD_600_ of the solution at the bottom of the biofilm in a 24-well plate. The results showed that the OD_600_ of the solution at the bottom of the FZB42 biofilm gradually increased with time after 72 h, while the OD_600_ of the solution at the bottom of the SZZ06 and SZZ18 biofilms remained relatively stable between 0 and 120 h (Figure 3B). The OD_600_ change curve of SZZ15 had a tendency to return to the wild type. This finding suggests that *ccdC* deficiency inhibits the release of cells from FZB42 biofilms.

The downward movement of bacteria in the biofilm may be affected by factors such as gravity, and thus, nonactive movement occurs. To rule out this possibility, we used another experimental approach, namely, the formation of a second ring on top of the original biofilm [20]. FZB42 first forms a biofilm at the solid—gas interface at the bottom of the test tube, and then liquid LBGM medium is added on top of it. The free cells form a biofilm at the liquid—gas interface, leaving a ring of ‘purple rings’; however, SZZ06 and SZZ18 have almost no biofilm (Figure 3C). The SZZ06 and SZZ18 bacteria in biofilms barely free themselves in an active way.

To further verify the role of *ccdC* in biofilm dispersal, we continued to incubate the biofilm until the final stage of dispersal. The dispersion of the SZZ18 biofilm is similar to that of SZZ06, showing defects in biofilm dispersion, and the SZZ15 phenotype showed some recovery to that of the wild type (Figure 3D,E). Taken together, these results suggest that *ccdC* is necessary for biofilm dispersal.

### 2.4. ccdC Regulates Sporulation and Motility

**Figure 4:**
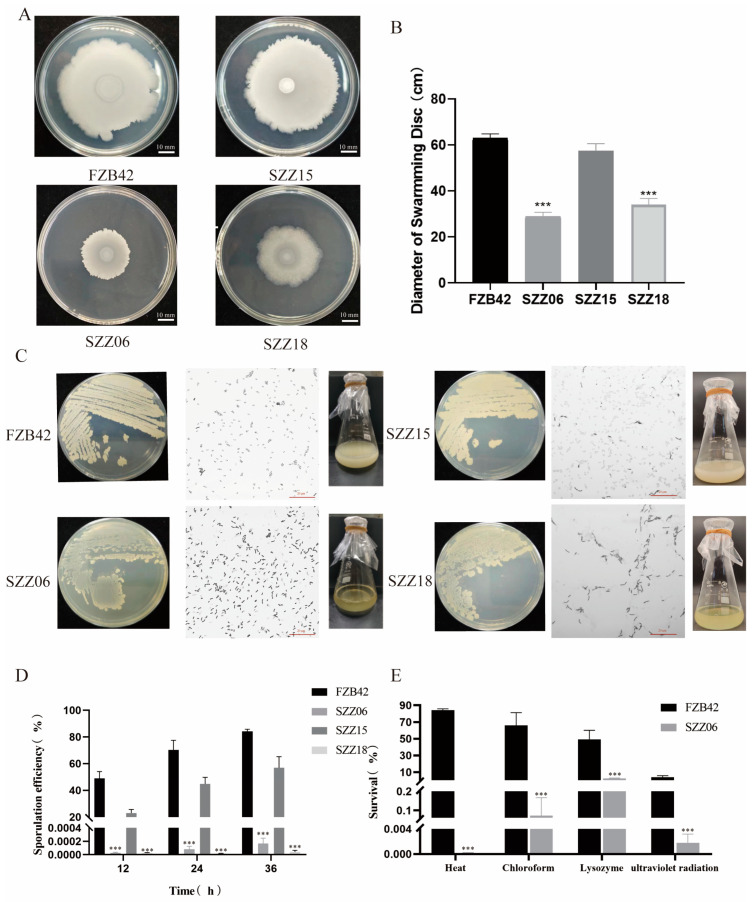
*ccdC* regulates swarming motility and sporulation. (**A**) Comparison of the swarming ability of FZB42, SZZ06, SZZ15, and SZZ18. (**B**) Diameter of swarming of FZB42, SZZ06, SZZ15, and SZZ18 collected from (**A**). (**C**) Morphological observation of FZB42, SZZ06, SZZ15, and SZZ18 on LB plates, an optical microscope, and DSM medium. (**D**) Spore formation rate of FZB42, SZZ06, SZZ15, and SZZ18 at 12 h, 24 h, and 36 h. (**E**) Spore formation rate of FZB42 and SZZ06 treated with high temperatures, chloroform, lysozyme, and ultraviolet light. *t*-test was performed, where *** *p* < 0.001.

It is widely accepted that robust motility is essential for bacteria to disengage and disperse from biofilms [23,24,25,26]. Prior to biofilm dispersal, a subset of bacteria within the biofilm typically engage in swimming, swarming, twitching, or floating within the constrained environment before exiting the biofilm [8]. We sought to investigate whether the *ccdC* gene influences motility. Hence, we evaluated swarming motility in strains FZB42, SZZ06, SZZ15, and SZZ18 (Figure 4A,B). SZZ06 and SZZ18 demonstrated a noticeable deficiency in swarming compared to FZB42, whereas the swarming capability of SZZ15 returned to wild-type levels (Figure 4A), indicating the significance of *ccdC* in swarming motility. Diminished motility likely impacts biofilm dispersal.

Biofilm maturation and sporulation are intricately intertwined; endospores typically form at the apex of the so-called fruiting bodies in mature biofilms [27,28]. Several genes implicated in spore formation undergo activation during biofilm maturation [29]. To assess spore production, FZB42, SZZ06, SZZ15, and SZZ18 bacterial colonies grown for 5 days on LB plates were stained with crystal violet. Microscopic examination revealed nearly complete phaseolus spore formation in the FZB42 and SZZ15 populations, whereas phaseolus endospores were notably absent in the SZZ06 and SZZ18 populations (Figure 4C). Additionally, cells from the FZB42, SZZ06, SZZ15, and SZZ18 populations were cultured in DSM media for 12 h, 24 h, and 36 h, and sporulation efficiency was assessed through heat treatment (Figure 4D). Remarkably, a low sporulation efficiency was observed in the SZZ06 cells at all points. SZZ18 exhibited a similar decline in sporulation efficiency, while SZZ15 displayed some degree of recovery compared to the wild type. The DSM media inoculated with FZB42, SZZ06, SZZ15, and SZZ18 continued to grow at 37 °C for 48 h. At this juncture, the FZB42 and SZZ15 cultures appeared milky white and turbid, whereas those of SZZ06 and SZZ18 showed clarification due to partial cleavage, mirroring observations on the LB plates (Figure 4C).

In addition to their remarkable heat resistance, bacteria also demonstrate robust resilience to ultraviolet light and noxious chemicals. Thus, in this investigation, lysozyme, chloroform, and ultraviolet light were employed to treat FZB42 and SZZ06 cultures cultivated for 36 h in DSM medium. The findings revealed that FZB42 displayed considerable tolerance, whereas SZZ06 exhibited persistently low survival rates, corroborating the outcomes of the heat treatment assay (Figure 4E). Collectively, these experimental findings suggest that *ccdC* gene deletion impairs the spore-forming capacity of FZB42. *ccdC* plays a critical role in regulating both spore formation and motility.

### 2.5. ccdC Modulates c-di-GMP Levels to Regulate Biofilm Dispersal

c-di-GMP stands out as one of the most prevalent and pivotal secondary messengers in bacteria, playing a crucial role in governing diverse cellular processes, including the cell cycle, cell differentiation, motility, biofilm formation, and virulence [7,30,31,32]. Notably, c-di-GMP-mediated signaling emerges as a pivotal regulator, particularly in Gram-negative bacteria, orchestrating the transition from planktonic to biofilm lifestyles [24,25]. The synthesis of c-di-GMP is intricately controlled by both diguanylate cyclases (DGSs), housing the GGDEF domain, and phosphodiesterases (PDEs), harboring either an EAL or HD-GYP domain. While DGCs catalyze c-di-GMP synthesis, PDEs facilitate its degradation. The coordinated activities of DGCs and PDEs finely tune the level of c-di-GMP, thus exerting precise control over bacterial behaviors and population dynamics [7,33,34].

**Figure 5:**
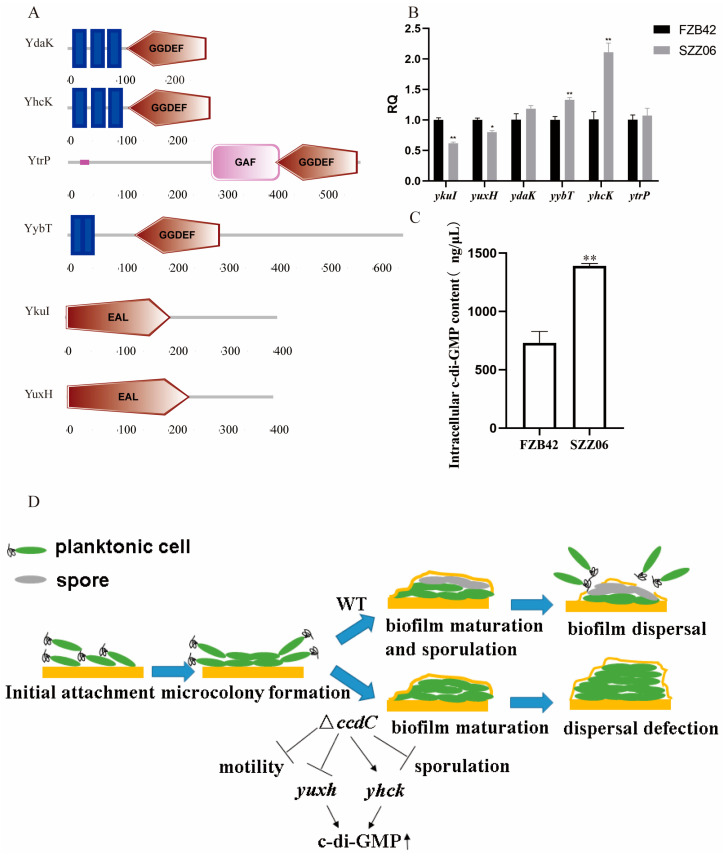
*ccdC* modulates c-di-GMP levels to regulate biofilm dispersal. (**A**) Domain prediction of genes associated with the synthesis and degradation of c-di-GMP. (**B**) c-di-GMP expression of genes related to synthetic degradation. (**C**) Intracellular c-di-GMP content of FZB42 and SZZ06. (**D**) The model for *ccdC*-regulated biofilm dispersion. *t*-test was performed, where * *p* < 0.05, ** *p* < 0.01.

The intricate involvement of c-di-GMP-mediated signaling in biofilm dispersal has been extensively explored across various Gram-negative bacterial species. Elevated c-di-GMP levels foster biofilm formation by stimulating the synthesis of adhesins and matrix polysaccharides, concurrently suppressing diverse forms of cellular motility [27,35,36]. Conversely, diminished c-di-GMP levels promote biofilm dispersal by enhancing cellular motility and extracellular (eDNA) production while reducing extracellular polysaccharide production, cell dimensions, and aggregation [37,38]. Furthermore, investigations into the role of c-di-GMP signaling in motility and biofilm formation within Gram-positive *Bacillus* spp. have been conducted [39,40,41,42]. Intriguingly, the presence of this signaling pathway in *B. velezensis* FZB42 piqued our interest. Initially, we employed the NCBI database (http://www.ncbi.nlm.nih.gov/Complete_Genomes/SignalCensus.html, accessed on 16 August 2023) to predict genes within the *B. velezensis* FZB42 genome potentially associated with this signaling cascade. Predictions from this database revealed that *B. velezensis* FZB42 encodes a total of four GGDEF domain-containing proteins (YdaK, YhcK, YtrP, and YybT) and two EAL domain-containing proteins (YkuI and YuxH). Utilizing SMART [43], we further predicted the structural domains of YdaK, YhcK, YtrP, YybT, YkuI, and YuxH, confirming the presence of GGDEF domains in YdaK, YhcK, YtrP, and YybT and EAL domains in both YkuI and YuxH (Figure 5A). Subsequently, we assessed the relative expression levels of *ydaK*, *yhcK*, *ytrP*, *yybT*, *ykuI*, and *yuxH* in FZB42 and SZZ06. Disruption of the *ccdC* gene in SZZ06 led to an elevated expression of the c-di-GMP synthase genes *ydaK*, *yhcK*, *ytrP*, and *yybT*, alongside a reduced expression of genes encoding the c-di-GMP-degrading enzymes YukI and YuxH (Figure 5B). Moreover, the enzyme-linked immunosorbent assay (ELISA) results revealed a significantly higher intracellular c-di-GMP concentration in SZZ06 compared to FZB42 (Figure 5C).

Finally, we present a model delineating how *ccdC* governs biofilm dispersion via the second messenger c-di-GMP (Figure 5D). Initially, planktonic cells adhere at the two-phase interface, leading to the formation of microcolonies. Subsequently, wild-type FZB42 progresses to spore formation and the development of mature biofilms. Ultimately, biofilm dispersal ensues as planktonic cells detach from the biofilm to explore new ecological niches, completing the biofilm life cycle process. However, the absence of *ccdC* dramatically diminished spore formation and motility while also impacting c-di-GMP synthesis and degradative enzyme-encoding gene expression, resulting in elevated c-di-GMP levels that impede biofilm dispersal. Notably, c-di-GMP is involved in dynamically regulating bacterial motility through concentration shifts [44], whereby heightened c-di-GMP levels suppress cell motility and, consequently, impede biofilm dispersion. Through its modulation of c-di-GMP levels, ccdC orchestrates the precise regulation of biofilm dispersal.

## 3. Discussion

Biofilms, intricate communities of surface-associated microorganisms enveloped within a self-generated extracellular matrix, represent a nearly ubiquitous trait among bacteria, thriving on a spectrum of natural and man-made surfaces [45]. Proficiency in biofilm formation holds paramount importance for PGPR to efficaciously colonize the rhizosphere and exert beneficial effects [46,47]. Al-Ali’s investigation underscored the pivotal role of biofilm formation in dictating tomato rhizosphere colonization by *B. velezensis* FZB42 [48], while Weng’s strategy of enhancing biofilm formation through *abrB* gene knockdown in *B. velezensis* SQR9 amplified root colonization and biocontrol efficacy [49]. While the correlation between biofilm-forming capacity and PGPR function has been extensively acknowledged, the nexus between the biofilm lifecycle, particularly biofilm dispersion, and PGPR function remains relatively unexplored. Hence, we opted to scrutinize the biofilm dispersion regulatory gene *ccdC* in a representative PGPR strain, *B. velezensis* FZB42, via transposon library screening to unravel the intricacies of biofilm dispersion regulation. Our investigations revealed discernible impacts on biofilm development in the ccdC-deficient transposon mutant SZZ06 (Figure 1), further validated through experiments involving ccdC knockout and complemented strains (Figure S1).

Presently, the regulation of biofilm dispersion in Gram-positive bacteria remains notably understudied. In B. cereus, the introduction of in vitro-synthesized autoinducer-2 (AI-2) has been shown to impede biofilm formation while promoting the liberation of cells from mature biofilms [50]. Similarly, in *B. subtilis* biofilms, another communication or dispersal molecule has been elucidated. As *B. subtilis* biofilms age, D-amino acids are produced just before their disassembly [51]. D-amino acids are effective against biofilms of *Staphylococcus aureus* and *P. aeruginosa*, so the production of D-amino acids by bacteria can be a common signal for biofilm disassembly [28]. The exploration of Gram-positive bacterial biofilms has mainly focused on signaling molecules, and there is a lack of knowledge about their regulatory mechanisms.

In *B. velezensis* FZB42, the gene *ccdC* remains unannotated, with no known functional characterization reported to date. Schiott previously identified the cytochrome c synthesis gene *ccdA* [52], prompting our hypothesis that *ccdC* might be associated with cytochrome synthesis. Through bioinformatics analysis, we predicted CcdC to be a hydrophobic, transmembrane protein, with subsequent subcellular localization studies indicating its likely presence in the cell membrane. Exploring its interaction network revealed potential associations with genes involved in nucleotide metabolism, spore formation, and biofilm regulation. Subsequent verification experiments demonstrated a significant reduction in spore production capacity in the SZZ06 mutant. In the process of studying SZZ06, we found a defect in the dispersion of its pellicles, which helps support the role of *ccdC* in regulating biofilm cycles, especially biofilm dispersion. Masaki Nishikawa reported that biofilm dispersal was synergistically caused by two mechanisms: a decreased expression of the *epsA* operon encoding exopolysaccharide synthetases and the induction of sporulation in *B. subtilis* [53]. In our research, we found that *ccdC* collaboratively regulates spore formation; this may be one of the ways in which *ccdC* regulates biofilm dispersion.

The absence of *ccdC* not only impaired swarming behavior in FZB42 but also contributed to the structural integrity of the biofilm. A growing body of experimental evidence suggests a close interplay between biofilm dispersion and motility [54,55,56]. It is widely acknowledged that decreased motility correlates with reduced biofilm dispersal propensity [20]. Our findings demonstrate that *ccdC* deficiency diminishes motility, aligning with this prevailing understanding.

c-di-GMP has emerged as a key regulator of biofilm dispersion in numerous Gram-negative bacteria, notably *P. aeruginosa* [57,58]. The induction of endogenous c-di-GMP triggers biofilm dispersal in Gram-negative bacteria [59]. While investigations into c-di-GMP-mediated biofilm dispersion in Gram-positive bacteria are limited, we posited whether analogous regulatory mechanisms exist in this bacterial group. In *B. subtilis*, c-di-GMP has been implicated in regulating biofilm development, sporulation, and motility [40]. Hull reported that c-di-GMP regulates spore formation in the Gram-positive strain *Streptomyces coelicolor* [60], and a significant relationship between c-di-GMP and sporulation and motility has been demonstrated in *B. subtilis* [61]. Collectively, these findings suggest a complex regulatory network intertwining motility, sporulation, biofilm formation, and dispersion in Gram-positive bacteria.

While direct evidence linking c-di-GMP to the regulation of biofilm dispersion in Gram-positive bacteria is lacking, our study reveals that the deficiency of *ccdC* in *B. velezensis* FZB42 leads to impaired sporulation and motility, accompanied by elevated c-di-GMP levels. Based on these findings, we propose the hypothesis that *ccdC*, a pivotal factor in FZB42 biofilm dispersion, co-regulates spore formation and motility by modulating intracellular c-di-GMP levels, thereby influencing biofilm dispersion dynamics.

## 4. Materials and Methods

### 4.1. Bacterial Strains and Growth Conditions

The bacterial strains used in this study are listed in Table 1. *B. velezensis* and *Escherichia coli* were grown at 37 °C in Luria Bertani (LB) medium solidified with 1.5% agar supplemented when necessary with the appropriate antibiotics (kanamycin at 5 μg/mL and erythromycin at 5 μg/mL). The LBGM contained 10 g of NaCl, 5 g of yeast extract, 10 g of tryptone, 1% glycerol, and 0.1 mM MnSO_4_ per liter. The solid LBGM contained 1.5% agar. The DSM medium contained 10 g of tryptone, 3 g of beef extract, 5 g of NaCl, 1 g of KCl, 0.12 g of MgSO_4_·7H_2_O, 1 mL of Ca(NO_3_), 10 μL of MnCl_2_, and 100 μL of 10 mM FeSO_4_ per liter.

### 4.2. Transposon Mutant Library

To generate a transposon library of *B. velezensis* FZB42, we followed the procedure described by Haldenwang [62]. The pMarA plasmid carrying the mariner-based transposon *TnYLB-1* was transformed into *B. velezensis* FZB42, which was named FBS239. FBS239 was cultured in LB broth (with 5 μg/mL erythromycin) at 30 °C and 200 rpm for 12 h. The bacterial fluid was spread on LB media supplemented with 5 μg/mL kanamycin at a dilution of 10^5^ and/or 10^6^. The plates were then treated at 50 °C for 12 h to select for transposants. Transformants were screened for plasmid-associated properties, i.e., kanamycin resistance at a permissive temperature for plasmid replication (30 °C) and kanamycin resistance at a restrictive temperature (50 °C) [63]. All single colonies were tested for km and Em resistance. A total of 5000 single colonies were selected to construct the transposon mutant library.

### 4.3. Mutant Status Verification

To identify transposon insertion sites, the mutant DNA was extracted by the CTAB method [64] and then digested by the restriction enzyme Taq I (Takara, Maebashi, Japan) at 65 °C for 15 min. The fragments were self-ligated by T4 ligase (Takara, Maebashi, Japan) at 16 °C. After purification, the primers FBO752 and FBO753 (Table 2) were used for inverse PCR. PCR products were sent for sequencing. By referring to the pMarA plasmid profile and FZB42 genomic DNA profile, the transposon insertion sites were identified.

### 4.4. Biofilm Development Assay

The strains were shaken overnight and then transferred (1%) to fresh LB media for continued incubation until the OD_600_ reached 1.0. Biofilm formation at the solid–air interface: 0.3 µL of cultures at a similar optical density were spotted onto an LBGM plate before being incubated at 25 °C for 48 h. Biofilm formation at the liquid—gas interface: 2 µL of cultures at a similar optical density were added to test tubes containing 5 mL of liquid LBGM before being incubated at 25 °C for 48 h. Biofilm development at the liquid—gas interface: 0.5 µL of cultures at a similar optical density were added to a 24-well plate containing 2 mL of liquid LBGM before being incubated at 25 °C for 120 h.

The bottom solution in the 24-well plate was drawn, and then the biofilms were eluted with methanol to test tubes every 12 h during the incubation of biofilms at the liquid—gas interface. The methanol was removed by centrifugation. The biofilm was dried until the weight did not change. The biofilm biomass was measured according to the dry weight of the biofilms.

### 4.5. Biofilm Dispersal Assay

The biofilm culture method refers to the above biofilm formation assay. The pellicles and the turbidity of the bottom solution under the pellicles were observed. Moreover, the OD_600_ of the culture medium under the biofilm was measured every 12 h.

The strains were shaken overnight and then transferred (1%) to fresh LB media for continued incubation until the OD_600_ reached 1.0. LBGM medium (1 mL) containing 2% agar was added to the bottom of the test tubes. One microliter of medium was inoculated on the surface of the solidified medium and incubated at 25 °C for approximately 140 h to form a biofilm at the gas—solid interface. Then, 3 mL of LBGM liquid medium was added along the wall of the test tube and incubated at 25 °C. Biofilm formation at the gas—liquid interface was observed every half hour. The medium between the gas—solid biofilm and the gas—liquid biofilm was removed, the biofilm was inverted in an oven for approximately 10 min, and the biofilm was fixed on the wall of the test tube at the gas—liquid interface. Crystal violet dye was added along the tube wall, and the tube was allowed to stand for 5 min. The crystal violet dye was removed, the test tubes were rinsed with distilled water, and the excess dye was removed by washing. The washed test tubes were placed in an oven and allowed to dry thoroughly so that the solid medium at the bottom fell off, after which images were recorded.

### 4.6. Swarming Motility Assay

The strains were shaken overnight and then transferred (1%) to fresh LB media for continued incubation until the OD_600_ reached 1.0. Ten microliters of the cultures were spotted in the center of LB plates containing 0.5% agar. Each treatment had three replicates. After incubation at 37 °C for 10 h at 70% humidity, the diameters of the colonies were measured.

### 4.7. Spore Formation Assay

The strains were shaken overnight and then transferred (1%) to fresh LB media for continued incubation until the OD_600_ reached 1.0. The cultures were transferred to fresh LB medium (1:100 dilution). After incubation for 12, 24, and 36 h, the cultures were heated at 85 °C for 20 min to kill the vegetative cells. CFUs were counted after the heat treatment, and the sporulation rate was referenced as the number of CFUs in the cultures that had not been heated at 85 °C.

DSM medium was used to promote spore formation. The cultures were transferred to fresh DSM media (1:100 dilution) and incubated at 37 °C and 200 rpm for 36 h to observe spore formation. The cultures were heated at 85 °C for 20 min and treated with chloroform, lysozyme, or ultraviolet radiation. CFUs were counted after these treatments, and the survival rate was referenced as the number of CFUs of the cultures that had not been treated with anything.

### 4.8. Construction of the ccdC Knockout Strain and Complement Strain

The *ccdC* sequence of FZB42 and its flanking regions were amplified with the primers szz38, szz39, and PrimeSTAR^®^ Max DNA Polymerase (Takara, Maebashi, Japan). The PCR product was added to a -A-tail using Taq DNA polymerase and then inserted into the commercial pMD-19 vector, resulting in the plasmid pMD-19-*ccdC*, which was named pSZZ11. The restriction endonuclease *Nru I* (Takara, Maebashi, Japan) was used to cut pSZZ11. The primers szz46 + *Nru I* and szz47 + *Nru I* were used to amplify the *speR* gene from pFB103. The *speR* gene was also cut by *Nru I* and then ligated to the pSZZ11 enzyme digestion product, yielding pSZZ17 (pMD-19-*ccdC*-*speR*), which was transformed into FZB42 as described previously. The transformants were verified with the primers szz01 and szz02, and the resulting strain was named SZZ15, the *ccdC* knockout strain.

The *ccdC* sequence of FZB42 and its flanking regions were amplified with the primers szz44 + *Kpn* I, *szz45* + *Cla I*, and PrimeSTAR^®^ Max DNA Polymerase (Takara, Maebashi, Japan). The PCR product and plasmid pFB01 [65] were both cut with *Cla I* and then ligated to obtain pSZZ13 (pFB01-*ccdC*), which was transformed into FZB42 as described previously. The transformants were verified correctly with the primers FBO550 and FBO16, which were named SZZ18, the *ccdC* complement strain.

### 4.9. Quantitative Real-Time Polymerase Chain Reaction (qRT—PCR)

The relative expression level of *ccdC* was determined in samples of *B. velezensis* FZB42 at intervals of 6 h. The expression of c-di-GMP synthetase and degrading enzyme-encoding genes was measured in samples of *B. velezensis* FZB42 and SZZ06 at 36 h. Total RNA was extracted and purified with TRIzol. Genomic DNA was removed with recombinant DNase I (TAKARA). The RNA concentration was calculated by measuring the absorbance at 260 nm, and 300 ng of RNA was used for the cDNA PrimeScript™ RT Master Mix Kit (Takara, Maebashi, Japan). qRT—PCR was carried out in a StepOnePlus Real-Time PCR System (Applied Biosystems, Waltham, MA, USA) using Power SYBR Green PCR Master Mix with the primers listed in Table 2. The conditions for qRT—PCR were as follows: initial denaturation at 95 °C for 10 min and 45 cycles of 15 s at 95 °C and 1 min at 60 °C. To confirm the specificity of the amplification products, melting curve analysis and agarose gel electrophoresis were performed to verify the presence of the targeted amplicons. The threshold cycle method (2^−ΔΔCT^) [66] was used to analyze changes in gene expression, and the data were normalized to the reference gene *gyrA*. For each sample, qRT—PCR was performed in triplicate, and the entire experiment was repeated twice with RNA samples extracted from independent cultures.

### 4.10. Intracellular c-di-GMP Measurement Assay

The strains were cultivated to OD_600_ = 0.8 and then ultrasonically crushed for analysis. The intracellular c-di-GMP level was measured with a cyclic diguanylate GMP (c-di-GMP) enzyme-linked immunosorbent assay (ELISA) kit (Bllswbio, Shanghai, China).

### 4.11. Statistical Analysis

All experiments were repeated in triplicate. Data between two groups were compared with a Student’s *t*-test with significance levels of * *p* < 0.05 and ** *p* < 0.01.

## Figures and Tables

**Table 1:** Strains used in this work.

Strain	Genotype	Description	Reference/Source
Plasmids			
	pMarA	pUC19 carrying *TnYLB-1* transposon mariner Himar1 transposase and promoters σA, KmR ApR EmR	Le Breton et al., 2006 [62]
	pMD-19	Commercial T-Vector (AmpR)	Takara, Maebashi, Japan
	pFB01	pVBF-*amyE::emR*, *gfp+*	Lab stock
	pFB103	pMD-19-*speR*	Lab stock
	pSZZ11	pMD-19-*ccdC*	This work
	pSZZ13	pFB01-*ccdC*	This work
	pSZZ17	pMD-19-*ccdC*-*speR*	This work
FZB42	Wild-type		Lab stock
SZZ06	FZB42 *ccdC::TnYLB-1*	Impaired in biofilm dispersal	This work
FBS239		pMarA → FZB42	Laboratory preservation
SZZ15	SZZ06 *amyE::ccdC*, *Emr*		This work
SZZ18	FZB42 *ccdC::speR*	pSZZ17 → FZB42	This work

**Table 2:** Primer sequences.

Primer	Sequence (5′ to 3′)
FBO752	GCTTGTAAATTCTATCATAATTG
FBO753	AGGGAATCATTTGAAGGTTGG
szz01	AAGCATCTAAAGTGCTGGAG
szz02	CAGATTGATCTTACTCCTTATC
szz38	ATCGGTCTTGCGTTTGCAG
szz39	TGTCCGGTCATATCAGTCAT
szz44 + *Kpn* I	CGGGGTACCATGATGATTATAATTTCATCCG
szz45 + *Cla* I	CCCATCGATATTCATCTGAATATCAGCGG
szz46 + *Nru* I	GTCTCGCGAGCATATGATCAGATCTTAAGGCC
szz47 + *Nru* I	GTCTCGCGATTGAAGCATGCAAATGTCACT
FBO16	TGGGTCAATCGAGAATATCGTC
FBO550	GTTTGTCTGCCGTGATGT
gyrA-fr	CCCACGTCCTCATAGTGACAG
gyrA-re	CGGACCGTTGCTGTCAGTGA
ydaK-fr	TGAAACAGCTGCGTGAAGAG
ydaK-re	AAGGAATTGCCGTAGCGTTC
yhcK-fr	AATCAGGCACACGATTGACG
yhcK-re	TTCGCTTTGTACAGCATCCG
ykuI-fr	CGCTGAGGAACAAAGAGTCG
yukI-re	CAATCTCCGCATCTGCCAAA
ytrP-fr	CGGAGCATAATGCGGTTCAT
ytrP-re	AGTGACGGCCTTTCATCAGA
yuxH-fr	TTCTTCACAGGGAGCTGGAG
yuxH-re	CGTCACAGTCCCAGTTGTTG
yybT-fr	GATAACTGGGTTGCCGCATT
yybT-re	TTTGACCAGGTGCTGATTGC

## Data Availability

Data are contained within the article.

## Supplementary Materials

The following supporting information can be downloaded at https://www.mdpi.com/article/10.3390/ijms25105201/s1.

## References

[1] Flemming H.C., Wingender J. (2010). The biofilm matrix. Nat. Rev. Microbiol..

[2] Hall-Stoodley L., Costerton J.W., Stoodley P. (2004). Bacterial biofilms: From the Natural environment to infectious diseases. Nat. Rev. Microbiol..

[3] Flemming H.-C., Wingender J., Szewzyk U., Steinberg P., Rice S.A., Kjelleberg S. (2016). Biofilms: An emergent form of bacterial life. Nat. Rev. Microbiol..

[4] Trejo M., Douarche C., Bailleux V., Poulard C., Mariot S., Regeard C., Raspaud E. (2013). Elasticity and wrinkled morphology of *Bacillus subtilis* pellicles. Proc. Natl. Acad. Sci. USA.

[5] Fröls S. (2013). Archaeal biofilms: Widespread and complex. Biochem. Soc. Trans..

[6] Fleming D., Rumbaugh K.P. (2017). Approaches to Dispersing Medical Biofilms. Microorganisms.

[7] Römling U., Galperin M.Y., Gomelsky M. (2013). Cyclic di-GMP: The First 25 Years of a Universal Bacterial Second Messenger. Microbiol. Mol. Biol. Rev..

[8] Petrova O.E., Sauer K. (2016). Escaping the biofilm in more than one way: Desorption, detachment or dispersion. Curr. Opin. Microbiol..

[9] Chua S.L., Tan S.Y.-Y., Rybtke M.T., Chen Y., Rice S.A., Kjelleberg S., Tolker-Nielsen T., Yang L., Givskov M. (2013). Bis-(3′-5′)-Cyclic Dimeric GMP Regulates Antimicrobial Peptide Resistance in Pseudomonas aeruginosa. Antimicrob. Agents Chemother..

[10] Chua S.L., Liu Y., Yam J.K.H., Chen Y., Vejborg R.M., Tan B.G.C., Kjelleberg S., Tolker-Nielsen T., Givskov M., Yang L. (2014). Dispersed cells represent a distinct stage in the transition from bacterial biofilm to planktonic lifestyles. Nat. Commun..

[11] Marks L.R., Davidson B.A., Knight P.R., Hakansson A.P. (2013). Interkingdom signaling induces Streptococcus pneumoniae biofilm dis-persion and transition from asymptomatic colonization to disease. mBio.

[12] Fleming D., Rumbaugh K. (2018). The Consequences of Biofilm Dispersal on the Host. Sci. Rep..

[13] Chowdhury S.P., Hartmann A., Gao X., Borriss R. (2015). Biocontrol mechanism by root-associated *Bacillus amyloliquefaciens* FZB42—A review. Front. Microbiol..

[14] Fan B., Wang C., Song X., Ding X., Wu L., Wu H., Gao X., Borriss R. (2018). *Bacillus velezensis* FZB42 in 2018: The Gram-Positive Model Strain for Plant Growth Promotion and Biocontrol. Front. Microbiol..

[15] Chen X.H., Koumoutsi A., Scholz R., Eisenreich A., Schneider K., Heinemeyer I., Morgenstern B., Voss B., Hess W.R., Reva O. (2007). Comparative analysis of the complete genome sequence of the plant growth–promoting bacterium *Bacillus amyloliquefaciens* FZB42. Nat. Biotechnol..

[16] López D., Fischbach M.A., Chu F., Losick R., Kolter R. (2009). Structurally diverse natural products that cause potassium leakage trigger multicellularity in *Bacillus subtilis*. Proc. Natl. Acad. Sci. USA.

[17] Banse A.V., Hobbs E.C., Losick R. (2011). Phosphorylation of Spo0A by the Histidine Kinase KinD Requires the Lipoprotein Med in *Bacillus subtilis*. J. Bacteriol..

[18] Huang Z., Wu L., Li X., Ma L., Borriss R., Gao X. (2020). Zn(II) suppresses biofilm formation in *Bacillus amyloliquefaciens* by inactivation of the Mn(II) uptake. Environ. Microbiol..

[19] Omer Bendori S., Pollak S., Hizi D., Eldar A. (2015). The RapP-PhrP Quorum-Sensing System of *Bacillus subtilis* Strain NCIB3610 Affects Biofilm Formation through Multiple Targets, Due to an Atypical Signal-Insensitive Allele of RapP. J. Bacteriol..

[20] Zhang Y., Qi J., Wang Y., Wen J., Zhao X., Qi G. (2022). Comparative study of the role of surfactin-triggered signalling in biofilm formation among different *Bacillus* species. Microbiol. Res..

[21] Bartolini M., Cogliati S., Vileta D., Bauman C., Rateni L., Leñini C., Argañaraz F., Francisco M., Villalba J.M., Steil L. (2019). Regulation of Biofilm Aging and Dispersal in *Bacillus subtilis* by the Alternative Sigma Factor SigB. J. Bacteriol..

[22] Fan B., Wang C., Ding X., Zhu B., Song X., Borriss R. (2019). AmyloWiki: An integrated database for *Bacillus velezensis* FZB42, the model strain for plant growth-promoting *Bacilli*. Database.

[23] Barraud N., Kjelleberg S., Rice S.A. (2015). Dispersal from Microbial Biofilms. Microbiol. Spectr..

[24] Guilhen C., Forestier C., Balestrino D. (2017). Biofilm dispersal: Multiple elaborate strategies for dissemination of bacteria with unique properties. Mol. Microbiol..

[25] Kaplan J.B. (2010). Biofilm Dispersal: Mechanisms, Clinical Implications, and Potential Therapeutic Uses. J. Dent. Res..

[26] Stacy A., Everett J., Jorth P., Trivedi U., Rumbaugh K.P., Whiteley M. (2014). Bacterial fight-and-flight responses enhance virulence in a polymicrobial infection. Proc. Natl. Acad. Sci. USA.

[27] Chai Y., Norman T., Kolter R., Losick R. (2010). An epigenetic switch governing daughter cell separation in *Bacillus subtilis*. Genes Dev..

[28] Abee T., Kovács T., Kuipers O.P., van der Veen S. (2011). Biofilm formation and dispersal in Gram-positive bacteria. Curr. Opin. Biotechnol..

[29] Vlamakis H., Aguilar C., Losick R., Kolter R. (2008). Control of cell fate by the formation of an architecturally complex bacterial community. Genes Dev..

[30] Yu M., Chua S.L. (2020). Demolishing the great wall of biofilms in Gram-negative bacteria: To disrupt or disperse?. Med. Res. Rev..

[31] Ha D.-G., O’Toole G.A. (2015). c-di-GMP and its Effects on Biofilm Formation and Dispersion: A *Pseudomonas Aeruginosa* Review. Microbiol. Spectr..

[32] Romling U., Galperin M.Y. (2017). Discovery of the Second Messenger Cyclic di-GMP. Methods Mol. Biol..

[33] McDougald D., Rice S.A., Barraud N., Steinberg P.D., Kjelleberg S. (2012). Should we stay or should we go: Mechanisms and ecological consequences for biofilm dispersal. Nat. Rev. Microbiol..

[34] Cai Y.-M., Hutchin A., Craddock J., Walsh M.A., Webb J.S., Tews I. (2020). Differential impact on motility and biofilm dispersal of closely related phosphodiesterases in *Pseudomonas aeruginosa*. Sci. Rep..

[35] Sprecher K.S., Hug I., Nesper J., Potthoff E., Mahi M.-A., Sangermani M., Kaever V., Schwede T., Vorholt J., Jenal U. (2017). Cohesive Properties of the *Caulobacter crescentus* Holdfast Adhesin Are Regulated by a Novel c-di-GMP Effector Protein. mBio.

[36] Nieto V., Partridge J.D., Severin G.B., Lai R.-Z., Waters C.M., Parkinson J.S., Harshey R.M. (2019). Under Elevated c-di-GMP in Escherichia coli, YcgR Alters Flagellar Motor Bias and Speed Sequentially, with Additional Negative Control of the Flagellar Regulon via the Adaptor Protein RssB. J. Bacteriol..

[37] Bassis C.M., Visick K.L. (2010). The Cyclic-di-GMP Phosphodiesterase BinA Negatively Regulates Cellulose-Containing Biofilms in *Vibrio fischeri*. J. Bacteriol..

[38] Yu S., Su T., Wu H., Liu S., Wang D., Zhao T., Jin Z., Du W., Zhu M.-J., Chua S.L. (2015). PslG, a self-produced glycosyl hydrolase, triggers biofilm disassembly by disrupting exopolysaccharide matrix. Cell Res..

[39] Fagerlund A., Smith V., Røhr K., Lindbäck T., Parmer M.P., Andersson K.K., Reubsaet L., Økstad O.A. (2016). Cyclic diguanylate regulation of *Bacillus cereus* group biofilm formation. Mol. Microbiol..

[40] Gao X., Mukherjee S., Matthews P.M., Hammad L.A., Kearns D.B., Dann C.E. (2013). Functional Characterization of Core Components of the *Bacillus subtilis* Cyclic-Di-GMP Signaling Pathway. J. Bacteriol..

[41] Yang Y., Li Y., Gao T., Zhang Y., Wang Q. (2018). C-di-GMP turnover influences motility and biofilm formation in *Bacillus amyloliquefaciens* PG12. Res. Microbiol..

[42] Fu Y., Yu Z., Liu S., Chen B., Zhu L., Li Z., Chou S.-H., He J. (2018). c-di-GMP Regulates Various Phenotypes and Insecticidal Activity of Gram-Positive *Bacillus thuringiensis*. Front. Microbiol..

[43] Schultz J., Milpetz F., Bork P., Ponting C.P. (1998). SMART, a simple modular architecture research tool: Identification of signaling domains. Proc. Natl. Acad. Sci. USA.

[44] Rumbaugh K.P., Sauer K. (2020). Biofilm dispersion. Nat. Rev. Microbiol..

[45] Stewart P.S., Franklin M.J. (2008). Physiological heterogeneity in biofilms. Nat. Rev. Microbiol..

[46] Yaryura P.M., León M., Correa O.S., Kerber N.L., Pucheu N.L., García A.F. (2008). Assessment of the Role of Chemotaxis and Biofilm Formation as Requirements for Colonization of Roots and Seeds of Soybean Plants by *Bacillus amyloliquefaciens* BNM339. Curr. Microbiol..

[47] Fan B., Borriss R., Bleiss W., Wu X. (2012). Gram-positive rhizobacterium *Bacillus amyloliquefaciens* FZB42 colonizes three types of plants in different patterns. J. Microbiol..

[48] Al-Ali A., Deravel J., Krier F., Béchet M., Ongena M., Jacques P. (2018). Biofilm formation is determinant in tomato rhizosphere colonization by *Bacillus velezensis* FZB42. Environ. Sci. Pollut. Res..

[49] Weng J., Wang Y., Li J., Shen Q., Zhang R. (2013). Enhanced root colonization and biocontrol activity of *Bacillus amyloliquefaciens* SQR9 by abrB gene disruption. Appl. Microbiol. Biotechnol..

[50] Auger S., Krin E., Aymerich S., Gohar M. (2006). Autoinducer 2 Affects Biofilm Formation by *Bacillus cereus*. Appl. Environ. Microbiol..

[51] Kolodkin-Gal I., Romero D., Cao S., Clardy J., Kolter R., Losick R. (2010). d-Amino Acids Trigger Biofilm Disassembly. Science.

[52] Schiött T., von Wachenfeldt C., Hederstedt L. (1997). Identification and characterization of the ccdA gene, required for cytochrome c synthesis in *Bacillus subtilis*. J. Bacteriol..

[53] Nishikawa M., Kobayashi K. (2021). Calcium Prevents Biofilm Dispersion in *Bacillus subtilis*. J. Bacteriol..

[54] Reuter M., Ultee E., Toseafa Y., Tan A., van Vliet A.H.M. (2020). Inactivation of the core *cheVAWY* chemotaxis genes disrupts chemotactic motility and organised biofilm formation in *Campylobacter jejuni*. FEMS Microbiol. Lett..

[55] Conrad J.C. (2012). Physics of bacterial near-surface motility using flagella and type IV pili: Implications for biofilm formation. Res. Microbiol..

[56] Du B., Gu Y., Chen G., Wang G., Liu L. (2020). Flagellar motility mediates early-stage biofilm formation in oligotrophic aquatic environment. Ecotoxicol. Environ. Saf..

[57] Pawar S.V., Messina M., Rinaldo S., Cutruzzolà F., Kaever V., Rampioni G., Leoni L. (2016). Novel genetic tools to tackle c-di-GMP-dependent signalling in *Pseudomonas aeruginosa*. J. Appl. Microbiol..

[58] Lin Chua S., Liu Y., Li Y., Jun Ting H., Kohli G.S., Cai Z., Suwanchaikasem P., Kau Kit Goh K., Pin Ng S., Tolker-Nielsen T. (2017). Reduced Intracellular c-di-GMP Content Increases Expression of Quorum Sensing-Regulated Genes in Pseu-domonas aeruginosa. Front. Cell. Infect. Microbiol..

[59] Andersen J.B., Kragh K.N., Hultqvist L.D., Rybtke M., Nilsson M., Jakobsen T.H., Givskov M., Tolker-Nielsen T. (2021). Induction of Native c-di-GMP Phosphodiesterases Leads to Dispersal of Pseudomonas aeruginosa Biofilms. Antimicrob. Agents Chemother..

[60] Hull T.D., Ryu M.-H., Sullivan M.J., Johnson R.C., Klena N.T., Geiger R.M., Gomelsky M., Bennett J.A. (2012). Cyclic Di-GMP Phosphodiesterases RmdA and RmdB Are Involved in Regulating Colony Morphology and Development in *Streptomyces coelicolor*. J. Bacteriol..

[61] Gao X. (2014). Exploration of C-di-GMP Signaling Pathway in *B. subtilis*. Ph.D. Thesis.

[62] Le Breton Y., Mohapatra N.P., Haldenwang W.G. (2006). In vivo random mutagenesis of *Bacillus subtilis* by use of TnYLB-1, a mariner-based transposon. Appl. Environ. Microbiol..

[63] Dietel K., Beator B., Budiharjo A., Fan B., Borriss R. (2013). Bacterial Traits Involved in Colonization of Arabidopsis thaliana Roots by *Bacillus amyloliquefaciens* FZB42. Plant Pathol. J..

[64] Möller E., Bahnweg G., Sandermann H., Geiger H. (1992). A simple and efficient protocol for isolation of high molecular weight DNA from filamentous fungi, fruit bodies, and infected plant tissues. Nucleic Acids Res..

[65] Fan B., Chen X.H., Budiharjo A., Bleiss W., Vater J., Borriss R. (2011). Efficient colonization of plant roots by the plant growth promoting bacterium *Bacillus amyloliquefaciens* FZB42, engineered to express green fluorescent protein. J. Biotechnol..

[66] Livak K.J., Schmittgen T.D. (2001). Analysis of relative gene expression data using real-time quantitative PCR and the 2-ΔΔCT method. Methods.

