# Supplementary material for: ccdC Regulates Biofilm Dispersal in Bacillus velezensis FZB42
Source: Int J Mol Sci. 2024 May 10;25(10):5201. doi: 10.3390/ijms25105201 (PMC11120784; doi:10.3390/ijms25105201)
Supplement: Supplementary file 1 [file ijms-25-05201-s001.zip › ijms-2939720-supplementary.pdf]

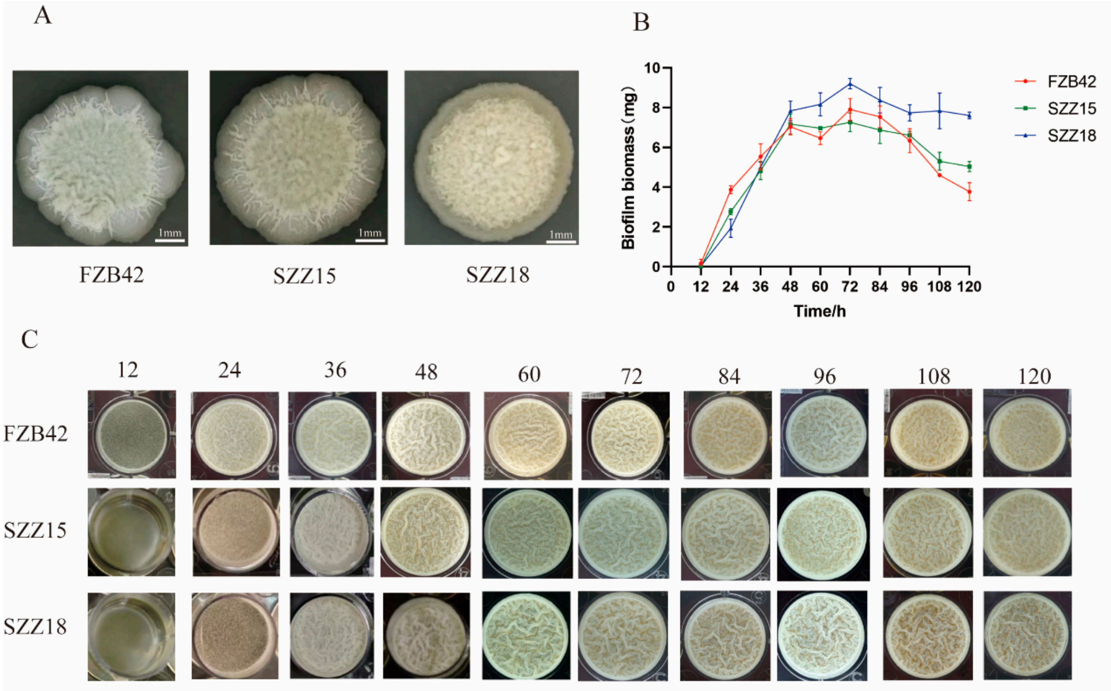

Figure S1. *ccdC* regulates biofilm development. (**A**) Biofilm formation of FZB42, SZZ15 and SZZ18 on solid surfaces ; (**B**) Dry weight of pellicle biofilms of FZB42, SZZ015 and SZZ18 collected from (**C**). (**C**) Pellicle biofilm development of FZB42, SZZ15 and SZZ18 over a period of 120 h.
